# Supplementary figures and images for: Quantitative proteomics analysis reveals important roles of N-glycosylation on ER quality control system for development and pathogenesis in Magnaporthe oryzae
Source: PLoS Pathog. 2020 Feb 24;16(2):e1008355. doi: 10.1371/journal.ppat.1008355 (PMC7058352; doi:10.1371/journal.ppat.1008355)

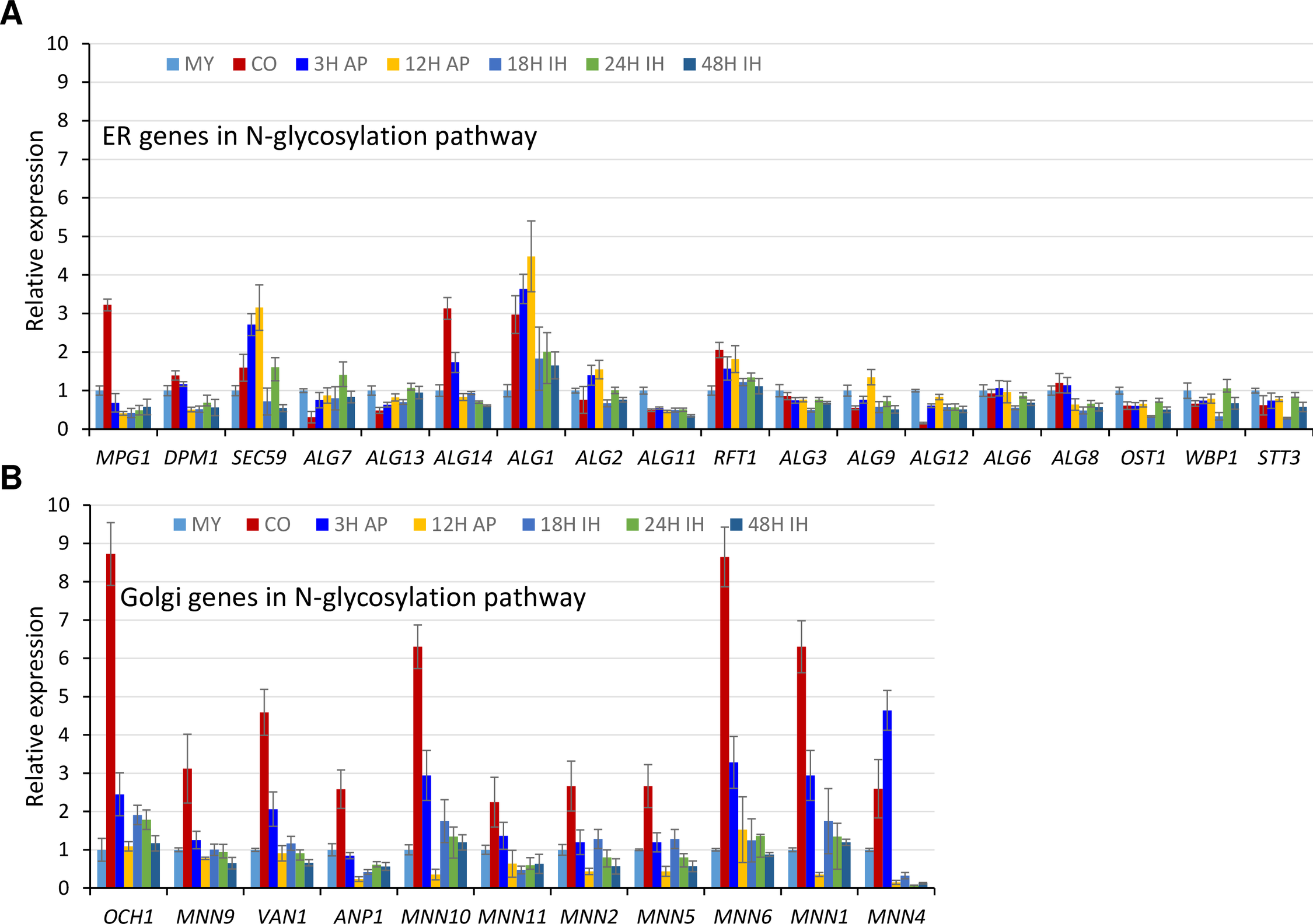

Supplement: S1 Fig — The phase specific expression of these genes was quantified by quantitative real-time PCR with synthesis of cDNA from each sample including mycelia, conidia, germ tubes, appressoria and invasive hyphae at indicated time points. Relative abundance was normalized by MoTub1. Three independent biological experiments with three replicates in each were performed. MY: mycelia; CO: conidia; AP: appressoria; IH: invasive hyphae. (TIF) [file ppat.1008355.s001.tif]

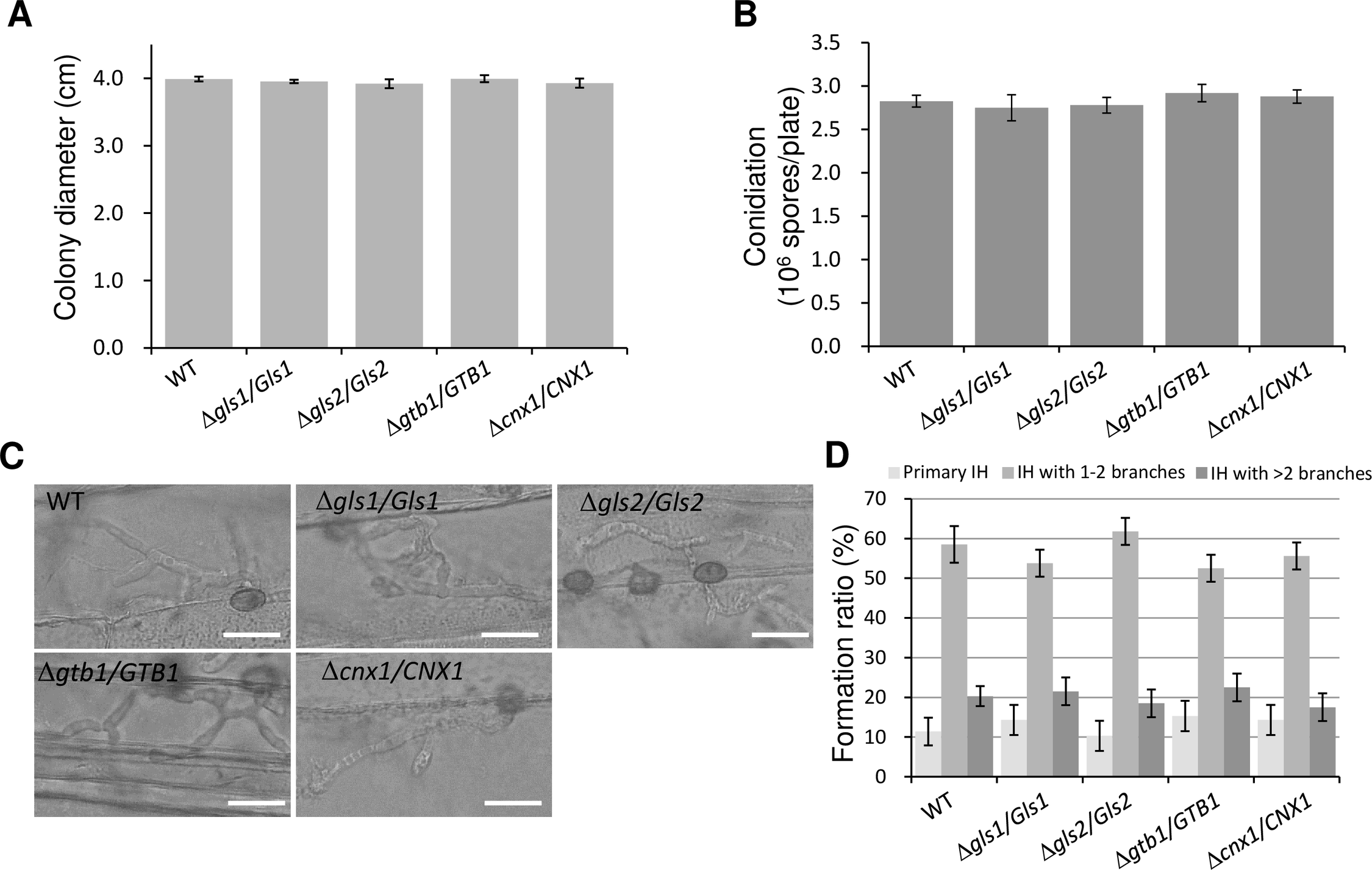

Supplement: S2 Fig — (A) Colony diameters of the wild-type (WT) and different complement strains. (B) Conidiation of the WT and different complement strains. (C) Invasive hyphae (IH) formed by the same set of strains in barley epidermal cells at 24 h post-inoculation (hpi). Bar, 20 μm. (D) Formed ratio of IH in barley epidermal cells at 24 hpi. (TIF) [file ppat.1008355.s002.tif]

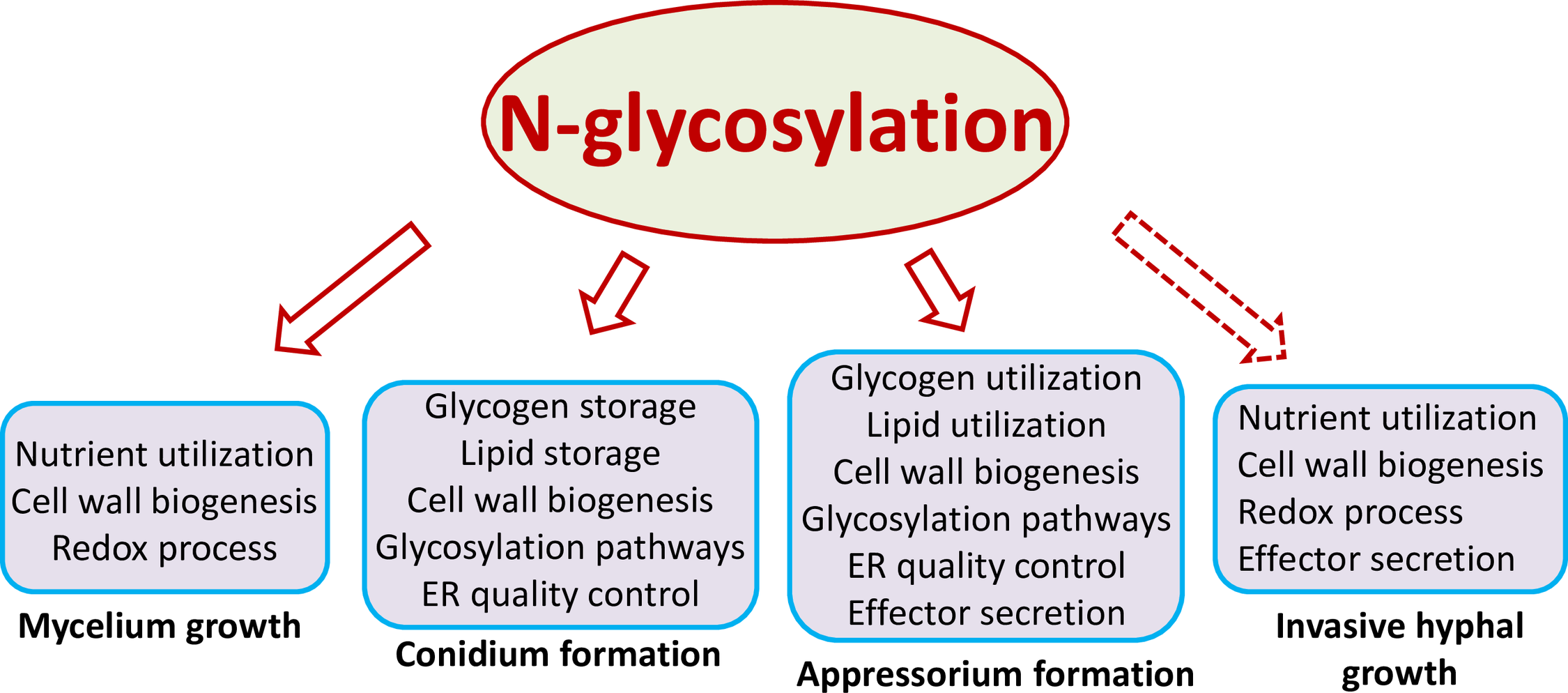

Supplement: S3 Fig — (TIF) [file ppat.1008355.s003.tif]
