# Supplementary material for: Quantitative proteomics analysis reveals important roles of N-glycosylation on ER quality control system for development and pathogenesis in Magnaporthe oryzae
Source: PLoS Pathog. 2020 Feb 24;16(2):e1008355. doi: 10.1371/journal.ppat.1008355 (PMC7058352; doi:10.1371/journal.ppat.1008355)
Supplement: Attachment — Submitted filename: Response letter.docx [file ppat.1008355.s010.docx]

**Response to reviewer 1**

Reviewer #1: The manuscript by Chen et al reports on quantitative N-glycoproteomics analysis to reveal systematic roles of N-glycosylation during fungal infection. The authors uncovered overall regulatory mechanisms of N-glycosylation at different development and infection stages of M. oryzae, and identified the endoplasmic reticulum quality control (ERQC) system were highly N-glycosylated, which is important for infection. They also proved N-glycosylation of the N497 site in ERQC component Gls1 was important for invasive growth. To my knowledge, this is the first time to uncover global roles of N-glycosylation during development and infection in the plant pathogenic fungi. Overall, this manuscript contains massive data and most of their conclusions are well supported. Here I list several points for consideration or suggestions for improving the manuscript.

Minor comments:

- For Fig. 8B, C and D, co-localization would be benefitted by the fluorescence intensity profiles.

**Response：**Thanks, the fluorescence intensity profiles have been added as suggested.

- Phenotypic analyses to the complementary strains of the ERQC genes should be provided as a supplemental data.

**Response：**Phenotypic analyses to the complementary strains have been provided in the supplemental Fig. 2.

- There are some spelling or grammatical errors can be found in the text, the authors need to comprehensively re-read the whole text to revise them. Some of these errors have been shown as follows.

- Page 2, line 27, ‘affect’ to ‘affected’.

- Page 3, line 39, ‘system’ to ‘systemic’.

- Page 4, line 54, ‘modifying’ to ‘altering’.

- Page 5, line 77, ‘had’ is not necessary.

- Page 5, line 94-95, two ‘which’ are used in one sentence, please refraise.

- Page 6, line 104, ‘were exist’ to ‘exist’.

- Page 6, line 110, ‘due to difficulty in separating’ to ‘due to difficulty separating’.

- Page 7, line 124, ‘in untreated’ to ‘without treatment’.

- Page 8, line 178, ‘on’ to ‘with’.

- Page 10, line 257, ‘involve’ to ‘involved’.

- Page 13, line 284, ‘to localize into’ to ‘to be localized into’.

- Page 14, line 299, ‘proved’ to ‘were proved’.

- Page 14, line 316, ‘both targets of’ to ‘both are targets of’.

- Page 17, line 391, ‘that corresponded’ to ‘corresponding’.

- Page 19, line 444, ‘stable’ to ‘stabilize’.

- Page 22, line 507, ‘peptides eluted and analyzed’ to ‘peptides were eluted and analyzed’.

**Response：**Thanks, all above were revised.

**Response to reviewer 2**

The manuscript defines the detectable set of N-linked glycosylated proteins from Magnaporthe oryzae mycelia, conidia, and germinated conidia that have produced appressoria. The function of N-glycosylation includes stabilizing protein structure and affecting other modifications, and its pattern may reflect the specialization of different cell types.

Here the authors have shown differential glycosylation patterns for the different cell types, that proteins involved in ER quality control are N-glycosylated, and that deletion of the ER quality control proteins leads to defective growth and development, and in growth in planta. One of these, gls-1 was assessed for the role of glycosylation and a change in the glycosylation at one site had little effect on growth of mycelia, a small effect on conidiation, but played a major role in growth in planta. This mutation appears to reduce stability of the protein so it may act as a knock down allele.

**Response：**Thanks a lot for all comments.

Results:

Biological importance of N-glycosylation at different development stages of M. oryzae

The first paragraph is weak. conA binds to glycoproteins, but also carbohydrates and glycolipids. Therefore staining of cell walls does not necessarily directly reflect glycoprotein level. If the authors were to provide evidence that carbohydrate and glycolipid levels are the same in cell walls of the different cell types, and the difference in staining is only due to glycoprotein binding, this would make sense.

**Response：**We agree with the reviewer’s concern, but we can’t find any suitable method to prove that carbohydrate and glycolipid levels are the same in cell walls of the different cell types. For instead, we specifically stained the GPI-anchored glycoproteins (the main N-glycoproteins in cell wall) with a Fluorescently Labelled Aerolysin (FLAER) dye (Alexa Fluor 488 proaerolysin). The FLAER can selectively bind to GPI-anchored glycoproteins on the anchor (Brodsky et al., Am J Clin Pathol, 2000, 114: 459–466), which is independent of N-glycan (Hong et al., EMBO J, 2002, 21: 5047–5056.) and exclude interference of carbohydrates and glycolipids. The result indicated that the FLAER staining showed a similar fluorescence level changes as detected by the ConA-FITC. There are also some other references used ConA-FITC to indirectly detect levels of the N-glycosylated glycoproteins. For example: Fauquenoy et al., 2008. Proteomics and glycomics analyses of N-glycosylated structures involved in Toxoplasma gondii. Mol Cell Proteomics. 7(5):891-910. Corresponding result was added in Line 118-124 (Fig. 1B).

The exposure time for Fig 1A is not provided in the methods or figure legend. Presumably these images would all have the same exposure time based on the author’s use of the images to inform the reader about their interpretation of glycoprotein level. However, if the exposure times are the same, why is the conidium cell wall in the conidium image stained very well, but the conidial cell wall does not stain at all with conA in the appressorium image?

**Response：**Thanks for the reminding. Yes, these images were obtained under the same property settings on the confocal microscope. We have added the settings and gain value in the methods, as shown in Line 496-499. However, we can’t tell why the intensities of the conidium cell wall at the conidium stage and appressorium stage are different, it could be an unrevealed mechanism during the appressorium formation. One possible explanation is that, an autophagic conidium cell death occurs during appressorium formation (Veneault-Fourrey et al., 2006. Autophagic fungal cell death is necessary for infection by the rice blast fungus), which may lead to disappear of the conidial cell wall glycoproteins.

It is not stated how the sample for conA staining of the infected cell was prepared. Is this a live cell and conA can enter the rice cell to stain the fungal cell wall or was the plant wall permeabilized in some way? I assumed the in vitro images are all live cells to which conA was added, but the protocol may have been different for the in planta experiment?

**Response：**Yes, the ConA-FITC stained a live cell. We observed the infection hyphae by dropping the conidia suspension onto the barley leaves as spore droplets. Before observing, we stained the infection hyphae by adding the ConA-FITC dye onto the spore droplets, after 30 min, then the barley epidermis was teared off for observation. Corresponding protocol was added in the method (Line 494-496).

This raises an interesting question. Spore tip mucilage provides a mechanism for surface attachment in spores. Whether spore tip mucilage is primarily carbohydrate or mixed carbohydrate and glycoprotein adhesive is not resolved. Surprisingly, spore tip mucilage is absent from image of the conidium in Fig 1A, why is that? Did conidia with spore tip mucilage have such a high level of fluorescence that the cell wall could not be visualized?

**Response：**Yes, the spore tip mucilage indeed existed in most conidia, but not all of the detected conidia. We provided a new photo to show the spore tip mucilage (Fig. 1B).

Would it be possible to extract the glycoprotein away from free carbohydrate to identity such glycoprotein(s) in spore tip mucilage? The authors are possibly thinking in this direction, so this work can lay the foundation for a future effort.

**Response：**Thanks for the suggestion. This is a really good idea, and we will try our effort to do this in the future.

It seems to me that Fig 1B is where the authors can should start and make the argument that there is an overall lower level of glycoproteins relative to actin in mycelia. There are five cell types assessed in Fig 1A, in vitro mycelia, conidia, germ tube, appressorium, and hyphae in planta. Although Fig1A does not address the amount of glycoprotein in the cell wall, it does clearly show the fundamentally different nature of cell walls in different cell types. The germ tube and appressorium have much stronger staining then the conidium.

**Response：**Thanks for the suggestion, we make Fig 1B as the start, and corresponding description order in the result was also changed in the text.

The statement that TM was used “to determine whether N-glycosylation is important for M. oryzae development and pathogenesis” is not very useful. We know a lot about glycosylation and the ER stress response. In other systems, with the dose used here, prolonged ER stress triggers programmed cell death. We don’t know from the methods or text how long TM treatment was except for the plant infection assay, where the treatment was just 6 hours long. Six hours is a very reasonable exposure time where one would expect ER stress induction and pro-survival signaling to be occurring. Beyond 24 hours, one might expect pro-death signaling to begin, hence, colony growth and other measures may be examining a different type of response than a shorter exposure. It seems much too simplistic to simply say this is a determination of whether N-glycosylation is important. We already know it is important. How is it important. The authors address this point later. But again, the effect of TM on colony growth may be a very different thing than how N-glycosylation is important for development and pathogenesis.

The effect of the 6 hours TM exposure on infection is relevant, however, DTT also induces ER stress, but does not directly affect glycosylation. Therefore, a critical control experiment that could easily have been done is absent. Is it glycosylation or ER stress that has an impact on these observations?

**Response：**Thanks for the reviewer’s explanation, we agree with the reviewer’s concerns, and thanks for the suggestions. In order to avoid those concerns,

1) the mycelia were treated with either 5 μg/ml TM or 10 mM DTT after 24 h incubation in liquid CM, and then continued to incubate for 12 h, and the mycelia dry weight was evaluated and the mycelial morphology was observed by CFW staining.

2) the conidia were produced by spreading mycelial fragments onto the OTA plates containing TM or DTT for conidiation, and the conidiophores were observed after 12h.

3) the infection hyphae were observed as before, just added the 10 mM DTT treatment as a control.

A new Figure 1 was provided. The result description was added in Line 135-138. Corresponding method was also added in Line 512-522.

Quantitative N-glycoproteomics profiling of different development stages in M. oryzae

The methods are described well except for the harvesting of the appressorium samples. Fig 2 shows the timing for when cultures were harvested but not the method. In the methods it is stated: “To perform quantitative N-glycoproteomics analysis, the mycelium, conidium and appressorium were harvested as mentioned above and were stored at −80°C before processing.”

It was frustrating that the harvesting method for appressoria was actually not mentioned above.

**Response：**The detailed method for harvesting appressorium samples has been added, as shown in Line 526-530: For appressoria collection, high concentration of the conidia suspension (2×10^6^ conidia mL^-1^) were sprayed onto a 21 cm×29.7 cm sized hydrophobic plastic surface and incubated in dark, moist plates at 28ºC. After 6 h, 12 h or 24 h, the appressoria were collected by scraping plastics surface and filtered with six layers lens wiping paper.

The finding of differential glycoprotein abundance in different tissues is consistent with Fig. 2B. However, there is a minor issue that bothers me. EMP1 was identified by homology to a Fusarium adhesive protein, and is a putative GPI-anchored, N-glycosylated, extracellular matrix protein expressed preferentially during appressorium development (Ahn et al. 2004. Mol Cells 17:166-173). EMP1 mutants have reduced appressorium formation and pathogenicity. Surprisingly, EMP1(MGG_00527) was not detected in this study. Perhaps the protein was not abundant enough, or for whatever reasons was not harvested (stuck to plastic) or unextractable (linked to cell wall). However, it would be nice to use this as an example in the discussion to alert the reader to potential limitations.

**Response：**Thank for the valuable suggestion, we thought this is really one nice example to alert the reader to potential limitations. We have added this in the discussion as shown in Line 428-440.

The following sections describe the findings of the N-glycoprotein analysis and will be very interesting to readers who are interested in the cell wall and ER function.

Sequence features of N-glycoproteome

Classification of identified N-glycoproteins

N-glycoprotein protein interaction networks

Comparing N-glycoproteins at different developmental stages of M. oryzae

The only issue here is that the time course of appressorium formation is described in Fig. 2 and mentioned a few times in the text but there is no real mention of analysis. Mycelia vs conidia vs appressoria (combined time course of appressorium formation) is discussed, but no discussion of 6 vs 12 vs 24 hours of conidiation ever appears. If there is no statistically significant difference between the 6, 12, and 24 hour time points, this is important for the reader to know. But I presume the authors expected there to be significant differences between these time points, so probably this should be discussed more than it is. The reader will be curious to know the authors’ explanation for the lack of differences between these.

**Response：**Thanks a lot for the suggestions. We added the analysis of differences between 6 vs 12 vs 24 hours, as well as the hyphae and conidia samples. Statistical analyses (principal component analysis (PCA), Pearson correlation heatmap, and clustering heatmap) showed that data from 6 h, 12 h and 24 h appressorium stages samples were similar, but clearly different from data of the mycelia and conidia samples (Fig. 4A-4C). Therefore, we combined time course data of appressorium formation. These analyses were added in the result and method, as shown in Line 227-237 and Line 583-588.

N-glycosylation regulates different cellular processes in development

N-glycosylation coordinates different glycosylation pathways during conidium and appressorium formation

Most proteins involved in ERQC were highly N-glycosylated in the conidium and appressorium

The concluding sentence of this section the authors say that ERQC is regulated by N-glycosylation. Since the title of the next section says this, maybe don’t need this sentence here.

**Response：**We have removed this sentence.

N-glycosylation regulates the ERQC system for fungal development and infection

It is an intriguing finding that there is differential glycosylation of proteins involved in ERQC during development. The glycosylation is required for stability and localization of the proteins. Is this differential glycosylation of ERQC components required for ERQC function or does it reflect the basal capacity for the response? Clearly ERQC must operate in all cell types. Is ER stress normally induced during appressorium formation? Does N-glycosylation regulate development or does development regulate N-glycosylation? The authors clearly show a relationship between development and ERQC and this is of interest to readers. Overall, the authors have done a good job in explaining the significance of the work.

**Response：**Thanks a lot for the comments.

DISCUSSION:

The question of N-glycosylation of proteins during infection is important. Presumably, during infection, the host environment is hostile and phytochemical activity, cell wall attack, reactive oxygen species, and other host strategies might induce stress along with the endogenous stress the fungus is undergoing via its rapid growth and deployment of pathogen strategies (chemical, protein, etc.). The pathogen is to be expected during infection. The manuscript is well written with only a few grammatical issues.

**Response：**Thanks for the comments.

There are some statements that are rationalizations. For example, starting at line 438, the statement that ERQC works at a low level in mycelia and a higher level in conidiation and infection where higher protein synthesis leads to unfolded proteins, inducing N-glycosylation of the proteins of the N-glycosylation pathway to allow them to more effectively glycosylate proteins to assist in the ERQC. Is that correct?

**Response：**This is really a prediction, so we deleted this description.

The statement on line 445. It is best left up to the reader to decide if the study provides deep insight. Some might have wanted to see the glycosylation patterns of proteins during growth in planta. The authors say that there is too small an amount of fungal material, however, I’m not sure this can be accepted at face value. Was an attempt made to detect the fungal N-glycosylated proteins in heavily infected leaf sheaths? It would be a deep insight to know if the fungus, after successfully overcoming the plant defense, is growing in an unstressed (like mycelia) or is always stressed (like appressoria) while growing in the plant.

**Response：**Thanks for the suggestion, we have changed the words to avoid self-evaluation. We agree with the reviewer’s comment that some might want to see N-glycosylation patterns of proteins in infection hyphae (IH). We actually have tried to collect the IH sample at 24-48 hpi, but no more than 1% of the biomass belongs to the fungal IH, while most of which belongs to the plant. Even we can detect the fungal N-glycosylated proteins in heavily infected host cells (necrotrophic growth stage), but this is not as important as the early invasive growth stage (biotrophic growth stage), and is not the theme of this study.

Again, it would be nice to have a discussion of known or expected glycoproteins and some discussion of the limitations of the study. How many glycoproteins might one expect to find in the N-glycoproteome (based on bioinformatics?) and how many were identified. Suggestions for improving the techniques.

**Response：**Thanks for the suggestions, we have revised as suggested. As shown in Line 428-440.

OVERALL this is an important study. The entry into the paper should emphasize Fig 1B and carefully interpret the meaning of the conA studies of Fig. 1A.

**Response：**Thanks for the suggestions, we have revised.

The emphasis on using the word regulation in describing the relationship between N-glycosylation and cell types should be more open to alternative explanations, for example N-glycosylation pathways might be regulated by signaling pathways that also regulate development. Both G-protein and cAMP pathway activation contribute to appressorium development. Does activation of those signaling pathways affect glycosylation levels? If the term regulation is to be used the authors should more clearly define what they mean by regulation.

**Response：**Thanks for the suggestions, we have checked and changed the words to avoid using of regulation. For those situations, we changed ‘regulate’ into ‘mediate’, which means an ‘indirect’ regulation and should be better.
